# Apathy, but not depression, predicts all-cause dementia in cerebral small vessel disease

**DOI:** 10.1136/jnnp-2020-323092

**Published:** 2020-07-10

**Authors:** Jonathan Tay, Robin G Morris, Anil M Tuladhar, Masud Husain, Frank-Erik de Leeuw, Hugh S Markus

**Affiliations:** 1 Department of Clinical Neurosciences, University of Cambridge, Cambridge, UK; 2 Department of Psychology, Kings College London, London, UK; 3 Department of Neurology, Donders Institute for Brain, Cognition and Behaviour, Radboud University Medical Center, Nijmegen, The Netherlands; 4 Nuffield Department of Clinical Neurosciences, University of Oxford, Oxford, UK

## Abstract

**Objective:**

To determine whether apathy or depression predicts all-cause dementia in small vessel disease (SVD) patients.

**Methods:**

Analyses used two prospective cohort studies of SVD: St. George’s Cognition and Neuroimaging in Stroke (SCANS; n=121) and Radboud University Nijmegen Diffusion Tensor and Magnetic Resonance Cohort (RUN DMC; n=352). Multivariate Cox regressions were used to predict dementia using baseline apathy and depression scores in both datasets. Change in apathy and depression was used to predict dementia in a subset of 104 participants with longitudinal data from SCANS. All models were controlled for age, education and cognitive function.

**Results:**

Baseline apathy scores predicted dementia in SCANS (HR 1.49, 95% CI 1.05 to 2.11, p=0.024) and RUN DMC (HR 1.05, 95% CI 1.01 to 1.09, p=0.007). Increasing apathy was associated with dementia in SCANS (HR 1.53, 95% CI 1.08 to 2.17, p=0.017). In contrast, baseline depression and change in depression did not predict dementia in either dataset. Including apathy in predictive models of dementia improved model fit.

**Conclusions:**

Apathy, but not depression, may be a prodromal symptom of dementia in SVD, and may be useful in identifying at-risk individuals.

## Introduction

Cerebral small vessel disease (SVD) is the leading vascular cause of dementia and plays a major role in cognitive decline and mortality.^1 2^ SVD affects the small vessels of the brain, leading to damage in the subcortical grey and white matter.^1^ The resulting clinical presentation includes cognitive and neuropsychiatric symptoms.^1^

Apathy is a reduction in goal-directed behaviour, which is a common neuropsychiatric symptom in SVD.^3^ Importantly, apathy is dissociable from depression,^3 4^ another symptom in SVD for which low mood is a predominant manifestation.^5^ Although there is some symptomatic overlap between the two,^6^ research using diffusion imaging reported that apathy, but not depression, was associated with white matter network damage in SVD.^3^ Many of the white matter pathways underlying apathy overlap with those related to cognitive impairment, and accordingly apathy, rather than depression, has been associated with cognitive deficits in SVD.^7^ These results suggest that apathy and cognitive impairment are symptomatic of prodromal dementia in SVD.

We tested the hypothesis that apathy, but not depression, predicts all-cause dementia using two independent cohorts of SVD patients: the St. George’s Cognition and Neuroimaging Study (SCANS) and Radboud University Nijmegen Diffusion Tensor and Magnetic Resonance Cohort (RUN DMC). We had three primary predictions: that baseline apathy, but not depression, predicts dementia after controlling for SVD-related cognitive impairment; that longitudinal changes in apathy, but not depression, would also predict dementia; and that the inclusion of apathy would improve dementia prediction models without apathy.

## Methods

### Participants

#### St. George’s Cognition and Neuroimaging in Stroke

Participants were recruited from stroke services at three UK hospitals covering a geographically contiguous region of South London. Included participants had a clinical lacunar stroke syndrome^8^ with MRI evidence of a lacunar infarct, defined as a high-signal lesion on diffusion-weighted imaging or cavitated lacune on T1-weighted imaging of diameter ≤1.5 cm, and confluent white matter hyperintensities (WMH) of Fazekas grade ≥2.^9^

Exclusion criteria were: stroke mechanisms other than SVD, including cortical infarcts, cardioembolism, intra/extracranial large artery stenosis >50%, or subcortical infarct diameter >1.5 cm; history of major neurologic or psychiatric condition excepting depression; non-fluent in English; unsuitable for MRI; or unable to give informed consent.

Baseline assessments were conducted a minimum of 3 months after the most recent stroke to reduce the influence of acute ischaemia on outcomes, with annual follow-up for up to 5 years. Participants provided written informed consent.

#### Radboud University Nijmegen Diffusion Tensor and Magnetic Resonance Cohort

Consecutive referrals to the Department of Neurology at Radboud University for symptoms of SVD between 2002 and 2006 were selected for possible participation.^10^ Included participants were between 50 and 85 years old and had evidence of cerebral SVD on neuroimaging, defined as lacunes or WMH.^11^ Individuals eligible because of a clinical lacunar stroke syndrome were included >6 months after the event to minimise the effect of the acute infarct on outcomes.

Exclusion criteria included: dementia, assessed using the Diagnostic and Statistical Manual of Mental Disorders (DSM)-IV-TR criteria^12^; Parkinson(-ism); intracranial haemorrhage; life expectancy <6 months; intracranial space-occupying lesion; disease interfering with cognitive testing or follow-up, including bipolar disorder and schizophrenia; current or recent use of acetylcholinesterase inhibitors, neuroleptic agents, levodopa or dopamine a(nta)gonists; WMH of non-vascular origin, such as multiple sclerosis; prominent visual or hearing impairment; language barrier and MRI contraindications or known claustrophobia. Participants provided written informed consent.

### Apathy and depression

#### St. George’s Cognition and Neuroimaging in Stroke

Apathy and depression were assessed using the 30-item Geriatric Depression Scale (GDS).^13^ This scale can be separated into a 6-item measure of apathy, with the remaining items assessing depression.^4^ One memory-related question (Do you feel you have more problems with memory than most?) was excluded in the calculation of the depression scores as this may bias assessments of dementia,^14^ leaving 23 items. The internal consistency of the apathy and depression subscales of the GDS, as measured by Cronbach's α, were adequate, (GDS_apathy_ α=0.63 and GDS_depression_ α=0.90). The psychometric characteristics of these GDS subscales have been explored in greater detail elsewhere.^4 14^

#### Radboud University Nijmegen Diffusion Tensor and Magnetic Resonance Cohort

Apathy was assessed using the 18-item clinician-rated Apathy Evaluation Scale (AES).^15^ The AES was only administered at 2011 and 2015, precluding an analysis of baseline AES scores. Furthermore, as data collection for RUN DMC is still ongoing, no data on progression to dementia were available beyond 2015, so only 2011 AES scores were analysed. Depression was assessed using the 20-item Center for Epidemiologic Studies Depression Scale (CESD),^16^ with two motivation-related items removed (felt that everything was an effort; could not get ‘going’). The internal consistency of the 18-item CESD (α=0.87) was not substantially different from the 20-item CESD (α=0.88).

### Cognitive assessment

Both studies administered neuropsychological tasks sensitive to processing speed (PS). PS deficits are a manifestation of vascular cognitive impairment and are associated with pathological white matter changes in SVD.^17^ Raw task scores were converted into z-scores using normative values and averaged to produce a composite measure of PS. Tasks in SCANS included the Digit Symbol Substitution Test, Grooved Pegboard Test, and the Speed of Information Processing task from the Brain Injury Rehabilitation Trust Memory and Information Processing Battery.^18^ Tasks in RUN DMC included the Paper-Pencil Memory Scanning Task and Letter Digit Substitution Task.^10^

### All-cause dementia diagnosis

#### St. George’s Cognition and Neuroimaging in Stroke

Dementia was defined using the DSM-5 definition of major neurocognitive disorder.^5^ Participants were diagnosed with dementia if they met one of the following criteria: dementia was diagnosed in a memory clinic or equivalent clinical service; panel consensus between a neurologist and clinical neuropsychologist that the clinical picture met DSM-5 criteria for dementia after blind review of medical records and cognitive assessments or Mini Mental State Examination (MMSE) <24, indicative of cognitive impairment,^19^ and an Instrumental Activities of Daily Living (IADL) ≤7, indicating reduced capabilities in daily living.^20^

The date of dementia was defined as the date of the diagnosis. If this was unknown, and the diagnosis was based on review of medical records or cognitive performance, the midpoint date between the visit at which the diagnosis was established and the previous visit was used.

#### Radboud University Nijmegen Diffusion Tensor and Magnetic Resonance Cohort

Dementia was defined using DSM-IV-TR criteria,^12^ which is broadly synonymous with the DSM-5 definition of major neurocognitive disorder,^21^ and was considered present if: dementia was diagnosed in a memory clinic or equivalent clinical service; panel consensus between a neurologist, clinical neuropsychologist and geriatrician that the clinical picture met DSM-IV-TR criteria for dementia after blind review of medical records and cognitive assessments; or MMSE <24 and IADL≤7.

The date of dementia was defined as the date clinical symptoms became compatible with the diagnosis. If this was unknown, the midpoint between the baseline visit and the date of the diagnosis was used, or failing this, the date of admission to a nursing home due to dementia.

### Statistical analysis

Statistical analyses were conducted using R 3.6.2 with the ‘survival’ package 3.1–8.^22^ All tests were two tailed with α=0.05. Analyses were conducted identically for SCANS and RUN DMC unless otherwise specified.

Clinical data were compared in four contexts:

1. Between SCANS and RUN DMC, assessing differences between datasets.
2. Between individuals who developed dementia or not within both datasets.
3. Between individuals who attended more than one assessment or not in SCANS, to see if any variables biased longitudinal assessments.
4. Between individuals stratified using median WMH scores in RUN DMC, to see if any variables differ based on disease severity.

Continuous variables were compared using Welch’s t-tests if normally distributed and Mann-Whitney U tests if not. Categorical or binary variables were compared using Pearson’s χ^2^.

To test the first hypothesis, which was that baseline apathy, but not depression, would predict dementia, multivariate Cox regression models were created with baseline apathy and depression scores, along with age, education, and PS as covariates (model 1). Event times were calculated from the first visit that apathy was assessed until the onset of dementia, death or the date of the most recent assessment.

To test the second hypothesis, which was that longitudinal change in apathy, but not depression, would predict dementia, we used a multivariate Cox model with all longitudinal observations in SCANS (model 2). Apathy and depression scores, as well as if the participant had developed dementia at that point, were allowed to vary between intervals. A participant-specific cluster variance was added as a term in the model to adjust for non-independent observations.

To test the third hypothesis, which was that the addition of baseline apathy scores would improve models predicting dementia, we compared two nested models. The first model included age, education and PS as covariates. The second model included the same terms as the first, but also added apathy. This second model was compared with the first model using a likelihood ratio test. A significant difference indicates that the second model explained more variance in outcomes than the first. We also calculated Akaike information criterion (AIC) scores for both models. The model with the lower AIC is the better fitting model.^23^

For all models, variance inflation factors for covariates <10, and proportionality of hazards verified by non-significant variable-level and model-level scaled Schoenfeld residual tests.^22^ Depression scores were log-transformed in both datasets due to positive skew. Cases with missing data in SCANS were listwise excluded (model 1: n=3; model 2: n=2).

## Results

### Study populations

#### St. George’s Cognition and Neuroimaging in Stroke

One hundred and tewnty-one participants were recruited at baseline, all of which were included in cross-sectional analyses. Of these 121, 18 completed only one assessment because of death (n=7), study withdrawal (n=6), relocation (n=1), lost to follow-up (n=2) or withdrawal from full neuropsychological testing (n=2), leaving 104 participants for the longitudinal analysis.

#### Radboud University Nijmegen Diffusion Tensor and Magnetic Resonance Cohort

Five hundred and three participants were recruited to the baseline assessment in 2006. Of these 503, 398 were able to attend follow-up in 2011. Reasons for missing the assessment included death (n=49), illness (n=19), relocation (n=5), lack of time (n=30) or lost to follow-up (n=2). An additional 46 were excluded due to reaching an endpoint before the 2011 assessment (n=15), or missing data (n=46), leaving 352 participants with complete data for the analysis. Due to our apathy measure not being administered in 2006, the 2011 follow-up will henceforth be referred to as the baseline for RUN DMC.

### Participant characteristics

Participants in SCANS had a higher burden of vascular disease compared with those in RUN DMC, evidenced by greater proportions of hypertension and hypercholesterolaemia (table 1). Participants in SCANS also showed lower IADL scores, indicating more impairment in activities of daily living.

**Table 1:** Characteristics of participants included for cross-sectional analysis in SCANS and RUN DMC

	SCANS (n = 121)	RUN DMC (n = 352)	P
Age	70.0 (9.7)	69.1 (8.2)	0.12
Sex, female (%)	43 (35.5)	142 (40.3)	0.41
Education			<0.001
Low (%)	56 (46.7)	34 (9.7)	
Medium (%)	43 (35.8)	198 (56.2)	
High (%)	21 (17.5)	120 (34.1)	
Hypertension (%)	112 (92.6)	283 (80.4)	0.003
Diabetes (%)	22 (18.2)	52 (14.8)	0.58
Hypercholesterolaemia (%)	104 (86.0)	175 (49.7)	<0.001
Smoking			0.003
Never (%)	48 (39.7)	103 (29.3)	
Ex (%)	49 (40.5)	205 (58.2)	
Current (%)	24 (19.8)	44 (12.5)	
BMI, kg/m^2^	27.0 (4.9)	27.8 (4.5)	0.023
MMSE	27.5 (2.7)	28.0 (2.2)	0.10
IADL	7.4 (1.2)	7.7 (1.0)	<0.001

BMI, body mass index; IADL, instrumental activities of daily living; MMSE, Mini Mental State Examination; RUN DMC, Radboud University Nijmegen Diffusion Tensor and Magnetic Resonance Cohort; SCANS, St. George's Cognition and Neuroimaging in Stroke.

### Baseline characteristics of participants who developed dementia

Follow-up data on progression to dementia was available for all participants. In SCANS, 24 of 121 participants (19.8%) developed dementia, while in RUN DMC, 38 of 352 participants (10.8%) developed dementia. Median time-to-event was 4.99 years (IQR=3.83–6.15) in SCANS and 3.33 (IQR=3.10–3.56) in RUN DMC. In both datasets, participants with dementia were characterised by higher apathy, but similar levels of depression at baseline (table 2).

**Table 2:** Baseline characteristics of participants who developed all-cause dementia

	SCANS			RUN DMC		
	Dementia (n = 24)	No dementia (n = 97)	P	Dementia (n = 38)	No dementia (n = 314)	P
Age	72.1 (10.5)	69.5 (9.5)	0.189	78.5 (4.5)	68.0 (7.8)	<0.001
Sex, female (%)	5 (20.8)	38 (39.2)	0.149	12 (31.6)	130 (41.4)	0.322
Education			0.043			0.002
Low (%)	12 (52.2)	44 (45.4)		9 (23.7)	25 (8.0)	
Medium (%)	11 (47.8)	32 (33.0)		23 (60.5)	175 (55.7)	
High (%)	0 (0.0)	21 (21.6)		6 (15.8)	114 (36.3)	
BMI, kg/m^2^	25.6 (5.7)	27.3 (4.6)	0.032	26.6 (5.9)	28.0 (4.3)	0.218
Hypertension (%)	23 (95.8)	89 (91.8)	0.804	30 (78.9)	253 (80.6)	0.982
Diabetes (%)	7 (29.2)	15 (15.5)	0.207	8 (22.2)	44 (14.6)	0.342
Hypercholesterolaemia (%)	23 (95.8)	81 (83.5)	0.219	27 (75.0)	148 (49.2)	0.006
Smoking			0.777			0.331
Never (%)	9 (37.5)	39 (40.2)		8 (21.1)	95 (30.3)	
Ex (%)	9 (37.5)	40 (41.2)		23 (60.5)	182 (58.0)	
Current (%)	6 (25.0)	18 (18.6)		7 (18.4)	37 (11.8)	
Apathy	3.6 (1.7)	2.8 (1.7)	0.047	35.6 (11.6)	26.6 (7.0)	<0.001
Depression	6.4 (6.0)	5.5 (5.1)	0.527	13.8 (5.8)	14.4 (4.0)	0.326
MMSE	24.9 (3.8)	28.2 (1.8)	<0.001	24.3 (3.5)	28.5 (1.4)	<0.001
PS index	−2.0 (0.4)	−0.8 (0.8)	<0.001	−1.9 (0.5)	−1.1 (0.7)	<0.001
IADL	6.4 (2.0)	7.7 (0.7)	<0.001	6.2 (2.1)	7.9 (0.5)	<0.001

BMI, body mass index; IADL, Instrumental Activities of Daily Living; MMSE, Mini Mental State Examination; PS, processing speed; RUN DMC, Radboud University Nijmegen Diffusion Tensor and Magnetic Resonance Cohort; SCANS, St. George's Cognition and Neuroimaging in Stroke.

### Longitudinal cohort characteristics in SCANS

In SCANS, 104 participants attended at least one follow-up assessment over the 5-year course of the study. Twenty individuals in the longitudinal cohort developed dementia (19.2%). Individuals who only attended the baseline were older and more cognitively impaired, but did not differ with regard to apathy and depression scores or dementia prevalence (table 3). ΔGDS_apathy_=−0.44 per year, while ΔGDS_depression_=−0.89 per year.

**Table 3:** Characteristics of participants with longitudinal data in SCANS

	Only baseline (n = 17)	Longitudinal cohort (n = 104)	P
Age	74.9 (8.0)	69.2 (9.8)	0.015
Sex, female (%)	7 (41.2)	36 (34.6)	0.802
Education			0.091
Low (%)	12 (70.6)	44 (42.7)	
Medium (%)	4 (23.5)	39 (37.9)	
High (%)	1 (5.9)	20 (19.4)	
Hypertension (%)	16 (94.1)	96 (92.3)	1.000
Diabetes (%)	5 (29.4)	17 (16.3)	0.339
Hypercholesterolaemia (%)	15 (88.2)	89 (85.6)	1.000
Smoking			0.436
Never (%)	9 (52.9)	39 (37.5)	
Ex (%)	6 (35.3)	43 (41.3)	
Current (%)	2 (11.8)	22 (21.2)	
BMI, kg/m^2^	27.7 (3.3)	26.9 (5.1)	0.327
Dementia	4 (23.5)	20 (19.2)	0.933
Apathy	3.0 (1.6)	2.9 (1.8)	0.982
Depression	4.9 (5.0)	5.8 (5.4)	0.500
MMSE	25.6 (3.1)	27.8 (2.5)	<0.001
PS index	−1.3 (1.0)	−0.9 (0.9)	0.074
IADL	6.9 (1.7)	7.5 (1.1)	0.075

BMI, body mass index; IADL, Instrumental Activities of Daily Living; MMSE, Mini Mental State Examination; PS, processing speed; SCANS, St. George's Cognition and Neuroimaging in Stroke.

### Disease severity group characteristics in RUN DMC

RUN DMC participants with WMH measurements (n=331) were divided into two groups using median WMH values (table 4). The above median WMH group was characterised by higher apathy, cognitive impairment and dementia prevalence, but did not differ in depression.

**Table 4:** Characteristics of participants in RUN DMC, stratified by median WMH scores

	Low severity (n = 166)	Moderate-high severity (n = 165)	P
Age	65.5 (6.7)	72.6 (8.1)	<0.001
Sex, female (%)	66 (39.8)	68 (41.2)	0.875
Education			0.009
Low (%)	9 (5.4)	21 (12.7)	
Medium (%)	88 (53.0)	97 (58.8)	
High (%)	69 (41.6)	47 (28.5)	
Hypertension (%)	122 (73.5)	143 (86.7)	0.004
Diabetes (%)	20 (12.7)	28 (17.5)	0.305
Hypercholesterolaemia (%)	70 (44.6)	91 (56.9)	0.038
Smoking			0.029
Never (%)	53 (31.9)	42 (25.5)	
Ex (%)	85 (51.2)	107 (64.8)	
Current (%)	28 (16.9)	16 (9.7)	
BMI, kg/m^2^	27.9 (4.4)	27.8 (4.8)	0.89
Dementia	5 (3.0)	28 (17.0)	<0.001
Apathy	25.5 (6.4)	29.4 (9.3)	<0.001
Depression	14.5 (3.6)	14.3 (4.7)	0.714
MMSE	28.6 (1.5)	27.6 (2.4)	<0.001
PS index	−1.1 (0.7)	−1.4 (0.7)	<0.001
IADL	7.9 (0.3)	7.5 (1.3)	<0.001

BMI, body mass index; IADL, Instrumental Activities of Daily Living; MMSE, Mini Mental State Examination; PS, processing speed; RUN DMC, Radboud University Nijmegen Diffusion tensor and Magnetic resonance Cohort; WMH, White matter hyperintensity volume.

### Cox regression analyses

Univariate Cox regression models were run for individual unadjusted covariates, followed by a multivariate model with all covariates (table 5). Model 1, which evaluated baseline apathy and depression scores in predicting dementia in both datasets, showed that higher apathy scores were associated with an increased dementia risk in SCANS, as were apathy scores in RUN DMC, after controlling for age, education and PS. In contrast, depression scores in both datasets were not associated with dementia in univariate or multivariate models.

**Table 5:** Cox proportional hazards models with all-cause dementia as the outcome variable

	SCANS				RUN DMC			
	Univariate		Multivariate		Univariate		Multivariate	
	HR (95% CI)	P	HR (95% CI)	P	HR (95% CI)	P	HR (95% CI)	
Model 1			C=0.904 (0.022)				C=0.914 (0.019)	
Apathy	1.30 (1.01 to 1.68)	0.041	1.49 (1.05 to 2.11)	0.024	1.09 (1.06 to 1.12)	<0.001	1.05 (1.01 to 1.09)	0.007
Depression	1.13 (0.70 to 1.85)	0.616	1.18 (0.59 to 2.37)	0.638	1.26 (0.89 to 1.77)	0.193	0.92 (0.64 to 1.31)	0.629
Age	1.03 (0.99 to 1.09)	0.159	1.07 (1.00 to 1.14)	0.047	1.17 (1.12 to 1.23)	<0.001	1.17 (1.11 to 1.24)	<0.001
Education	0.57 (0.30 to 1.07)	0.08	0.92 (0.42 to 2.03)	0.844	0.43 (0.26 to 0.71)	0.001	0.67 (0.39 to 1.17)	0.158
PS	0.10 (0.04 to 0.22)	<0.001	0.03 (0.01 to 0.12)	<0.001	0.16 (0.09 to 0.27)	<0.001	0.27 (0.14 to 0.54)	<0.001
Model 2			C=0.937 (0.027)					
Apathy	1.11 (0.86 to 1.42)	0.436	1.53 (1.08 to 2.17)	0.017				
Depression	1.29 (0.67 to 2.46)	0.443	0.65 (0.10 to 4.38)	0.654				
Age	1.05 (0.97 to 1.14)	0.231	1.21 (1.02 to 1.45)	0.033				
Education	1.14 (0.71 to 1.82)	0.586	4.00 (1.50 to 10.70)	0.006				
PS	0.10 (0.04 to 0.23)	<0.001	0.00 (0.00 to 0.03)	<0.001				

Raw depression scores were log-transformed due to positive skew in all analyses.

PS, processing speed; RUN DMC, Radboud University Nijmegen Diffusion Tensor and Magnetic Resonance Cohort; SCANS, St. George's Cognition and Neuroimaging in Stroke.

**Figure 1:**
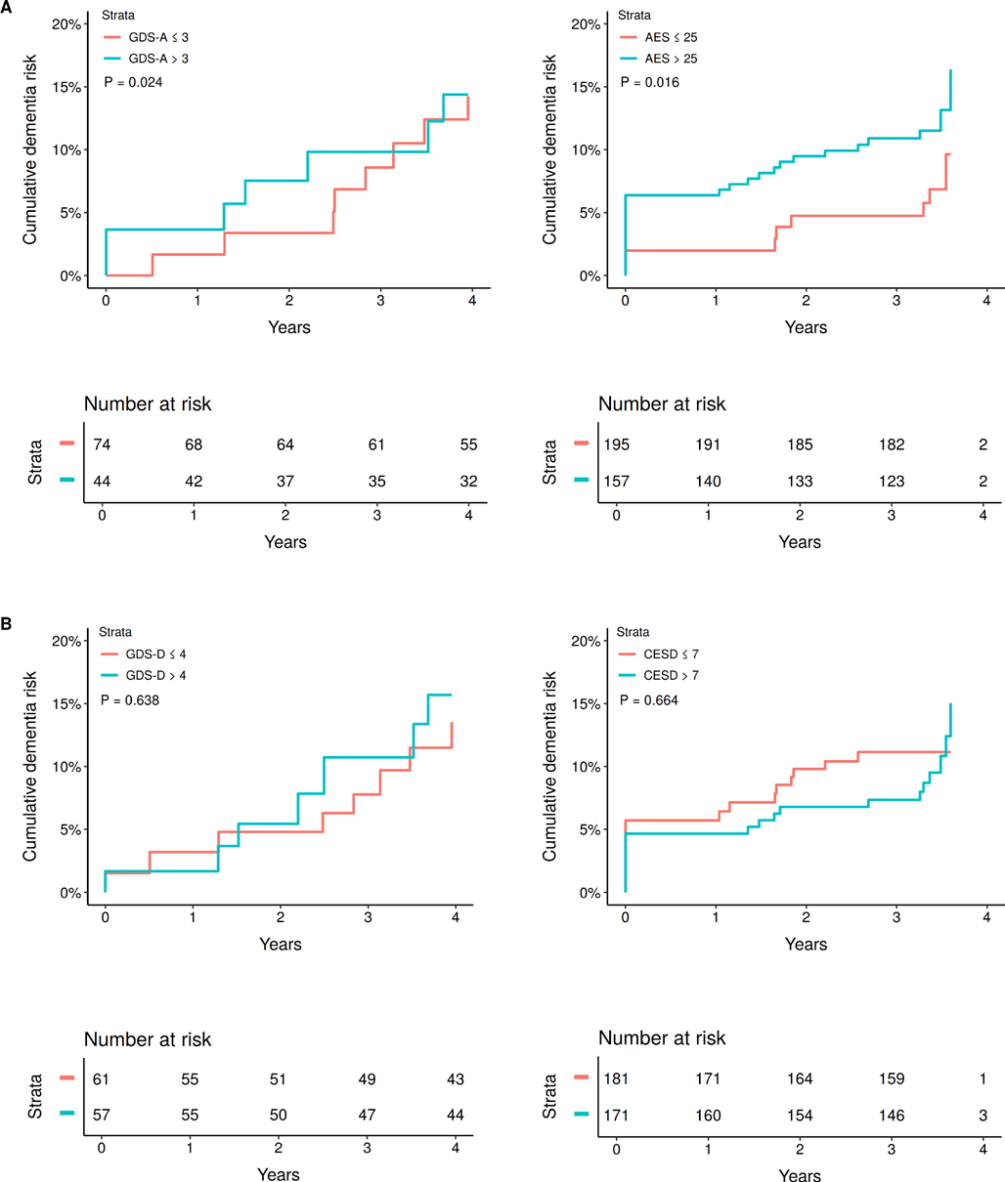
Cumulative dementia risk for participants stratified by median baseline apathy and depression scores. (A) Dementia risk for patients stratified on median apathy scores in scans (left) and run DMC (right); (B) dementia risk for patients stratified on median depression scores in scans (left) and run DMC (right). P values from Cox regression results. AES, Apathy Evaluation Scale; CESD, Center for Epidemiological Studies Depression Scale; DMC, Diffusion Tensor and Magnetic Resonance Cohort; GDS, Geriatric Depression Scale;

To illustrate the impact of apathy and depression on dementia risk, model 1 was rerun using median apathy and depression scores. Covariate-adjusted Kaplan-Meier survival curves demonstrated that higher apathy was associated with a greater dementia risk over time in SCANS and RUN DMC (figure 1A), while depression showed mixed results (figure 1B).

Results in model 1 were largely consistent for model 2, which analysed longitudinal apathy and depression in SCANS. Change in apathy, but not change in depression, was associated with greater dementia risk in the multivariate model. One important difference from the cross-sectional models was that change in apathy was not associated with dementia when assessed in a univariate model.

### RUN DMC results do not depend on disease severity

Given that disease severity groups in RUN DMC differed on key variables (table 4), we reran model 1 with the median WMH grouping variable as a covariate. Results remained consistent, with apathy being associated with dementia, (HR 1.06, 95% CI 1.02 to 1.10, p=0.002), but not depression, (HR 1.06, 95% CI 0.47 to 2.41, p=0.89). Age remained associated with dementia, (HR 1.17, 95% CI 1.09 to 1.24, p<0.001), as did PS, (HR 0.29, 95% CI 0.14 to 0.60, p<0.001). Education was not associated with dementia, (HR 0.63, 95% CI 0.34 to 1.15, p=0.13), nor was WMH group (HR 1.28, 95% CI 0.45 to 3.64, p=0.65).

### Apathy improves models predicting dementia

To evaluate whether the addition of apathy increased the utility of these models for predicting dementia, a model with age, education and PS as covariates was compared with a model with apathy, age, education and PS. Likelihood ratio tests revealed that the inclusion of apathy led to improved model fit in both SCANS (χ^2^=5.30, p=0.021) and RUN DMC (χ^2^=5.67, p=0.017). These models were also compared using the AIC. Models with apathy minimised showed lower AIC values than those without apathy in SCANS (AIC_no apathy_=153.4, AIC_with apathy_=146.8) and RUN DMC (AIC_no apathy_=332.0, AIC_with apathy_=327.5), indicating that models with apathy better fit the data.^23^

## Discussion

We tested the hypothesis that apathy, but not depression, is associated with dementia in patients with SVD. We found that higher baseline apathy, as well as increasing apathy over time, were associated with an increased dementia risk. In contrast, neither baseline depression or change in depression was associated with dementia. The relationship between apathy and dementia remained after controlling for other well-established risk factors including age, education and cognition.^24^ Finally, adding apathy to models predicting dementia improved model fit. These results suggest that apathy may be a prodromal symptom of dementia in patients with SVD.

Importantly, our hypotheses were investigated in two independent cohorts of symptomatic patients with MRI-confirmed SVD. These cohorts differed in overall disease burden as well as apathy and depression assessments, but had identical definitions for radiological markers of SVD.^11^ Despite these differences, we found that apathy, but not depression, was consistently associated with dementia risk in both studies. This suggests that our findings are robust and reproducible, and may be generalisable across a broad spectrum of SVD severity. SCANS, by virtue of its inclusion criteria, had a higher burden of SVD pathology when compared with RUN DMC, reflected in a higher proportion of vascular risk factors and greater IADL impairment. This may explain why SCANS had nearly double the dementia prevalence compared with RUN DMC despite similar dementia criteria and follow-up durations.

Intriguingly, we found that longitudinal change in apathy was not associated with dementia in a univariate model, but became significant in a multivariate model. This is an example of positive confounding, whereby effect sizes are overestimated due to a confounding variable.^25^ This effect may stem from a variety of factors, but may indicate that change in apathy is only predictive of dementia in a particular subgroup. Unfortunately, the sample size of the longitudinal cohort of SCANS was too small to investigate this possibility (ie, interaction effects), and must therefore be explored in future research with larger cohorts.

Our results initially appear to diverge with findings from the Prevention of Dementia by Intensive Vascular Care trial, which showed that apathy and depression, assessed using the 15-item GDS, predicted incident dementia in community-dwelling individuals.^14^ The investigators, however, found that the associations between depression and dementia were largely driven by the GDS question assessing memory complaints. After removing that item from the calculation of depression scores, which we did a priori, this association became non-significant. Furthermore, the authors also found that an interaction between apathy and a history of stroke predicted dementia. This interaction may have been partially driven by patients with SVD with lacunar stroke, which was explored in our study. Our results may therefore contextualise their findings in individuals with SVD.

Our findings may clarify why there are inconsistent reports of associations between late-life depression or depressive symptoms and dementia risk.^26^ Clinical depression scales may also assess apathy, as evidenced by motivation-related questions on the GDS and CESD. These apathy items may be a factor underlying the relationship between depressive symptoms and dementia in the elderly. This is an important consideration for future studies that use scales to measure depression. In our study, removing these apathy items had minimal effects on internal consistency, suggesting that this may be a valid approach for assessing a more theoretically narrow depression construct.

The number of people living with dementia worldwide is projected to triple by 2050,^27^ making early diagnosis and intervention increasingly important. Late-life cognitive functioning may be maintained by targeting modifiable factors such as cardiovascular risk, physical activity and diet.^28^ Our results support the notion that measuring apathy may be clinically useful as a non-invasive and inexpensive method for identifying patients at-risk for developing dementia.^14^ Additionally, our longitudinal findings suggest that continued monitoring of apathy may be a way to assess changes in dementia risk. Individuals identified as having high apathy, or increasing apathy over time, could be sent for a more detailed neurocognitive or neuropathological examination, or be selected for therapeutic interventions.

Examining relationships between apathy and dementia-related mortality is another important area for future research, given findings that apathy is associated with all-cause mortality.^29^ These results, in conjunction with ours, suggests that apathy may be associated with an increased risk of dementia-related mortality. If this is the case, then apathy may signal for a more severe prognosis in vascular dementia patients.

Our work also suggests that another area for research lies in identifying mechanisms linking apathy to dementia onset. Recent neuroimaging work suggests that similar white matter networks underlie motivation and normal cognitive function in SVD.^3^ It is possible that vascular pathology that damages these networks^30^ leads to a prodromal form of dementia which presents with apathy and cognitive deficits. Over time, SVD-related pathology increases, which is paralleled by increasing cognitive and motivational impairment,^31 32^ eventually becoming severe enough to meet criteria for a dementia state. This implies that apathy is not a risk factor for dementia per se, but rather an early symptom of white matter network damage. Indeed, recent theoretical work proposed that certain symptoms of apathy are synonymous with defined cognitive deficits.^33^ If this is the case, then apathy may manifest early as a reduction in attention towards reward stimuli, then later, as an inability to learn or remember rewarding behaviours. This would be consistent with initial executive deficits that are followed by declining episodic memory, which may be a cognitive phenotype of SVD patients that develop vascular or mixed dementia.^32 34^

There are some limitations to our study. Full follow-up data for dementia are not available in RUN DMC, precluding the longitudinal analysis of apathy conducted in SCANS. Another limitation was the use of clinical scales to assess apathy and depression. Although a structured clinical interview may have yielded a more accurate measure of neuropsychiatric symptomatology, this was not feasible due to time constraints. A related limitation was our use of the MMSE and IADL in detecting patients with dementia. Although the cut scores used have been determined empirically, many of the studies assessing this have used older DSM definitions. Further work will be needed to evaluate the accuracy of the MMSE and IADL at detecting major neurocognitive disorder using DSM-5 criteria.

Participant drop-out was a concern in both studies. It is possible that individuals with higher baseline apathy were less likely to attend follow-ups, potentially confounding results. While this was not the case in SCANS, this could not be confirmed in RUN DMC. This led to smaller sample sizes and fewer events in both populations, precluding comprehensive subgroup or interaction testing as mentioned earlier.

Finally, the time scale that participants were assessed on was relatively short, with data for both studies ending within 5 years. Preliminary evidence suggests that the relationships between apathy, depression and dementia may change over longer periods,^14^ and our results need be replicated over a longer duration.

Our work has shown that apathy, but not depression, predicted all-cause dementia in SVD, supporting the hypothesis that apathy is a prodromal symptom of dementia. This shows that distinguishing between these symptoms has implications for clinical practice and research. It also suggests that apathy may be useful in predictive models of dementia, and that the assessment of apathy over time may be informative for dementia diagnosis. Finally, it provides a basis for future studies attempting to understand mechanisms linking apathy, vascular cognitive impairment and dementia.
